# Eupatilin Alleviates Hyperlipidemia in Mice by Inhibiting HMG-CoA Reductase

**DOI:** 10.1155/2023/8488648

**Published:** 2023-06-21

**Authors:** Kyung-Joo Kim, Nam E. Kang, Yoon Sin Oh, Se-Eun Jang

**Affiliations:** Department of Food and Nutrition, Eulji University, 553, Sanseong-daero, Seongnam, Gyeonggi-do 13135, Republic of Korea

## Abstract

*Artemisia princeps* (family *Asteraceae*) is a natural product broadly used as an antioxidative, hepatoprotective, antibacterial, and anti-inflammatory agent in East Asia. In the present study, eupatilin, the main constituent of *Artemisia princeps*, was investigated as an antihyperlipidemic agent. Eupatilin inhibited 3-hydroxy-3-methylglutaryl (HMG)-CoA reductase (HCR), an enzyme that is a therapeutic target for hyperlipidemia, in an *ex vivo* assay using rat liver. In addition, oral administration of eupatilin significantly lowered the serum levels of total cholesterol (TC) and triglycerides (TG) in corn oil-induced and Triton WR-1339-induced hyperlipidemic mice. These results suggest that eupatilin can alleviate hyperlipidemia by inhibiting HCR.

## 1. Introduction

*Artemisia princeps* (AP) (family *Asteraceae*) is a natural product that is broadly used as an antioxidative, hepatoprotective, antibacterial, and anti-inflammatory agent in China, Japan, and Korea [1]. Eupatilin, the main component of AP, ameliorates gastric ulcers [2], motivates apoptosis of human gastric cancer cells [3], and suppresses the inflammatory response induced by carrageenan through the control of nuclear factor-kappa B (NF-*κ*B) [1].

Lipid metabolism homeostasis is maintained by a balance between lipid synthesis and degradation, and disruption in this balance can lead to hyperlipidemia [4]. The enzyme 3-hydroxy-3-methylglutaryl (HMG)-CoA reductase (HCR), a rate-limiting enzyme for the biosynthesis of cholesterol from acetate, is a therapeutic target for hyperlipidemia, and several HCR inhibitors have been developed [5]. Statins are HCR inhibitors widely used as a treatment for hyperlipidemia. Statins mediate their anti-inflammatory effects by inhibiting NF-*κ*B [6–8].

In addition, statins are effective in terms diabetic neuropathic pain through their anti-inflammatory effects. These effects of statins may be like those of N-acetylcysteine [9, 10]. However, although the anti-inflammatory effects of eupatilin have been reported [1], its hypolipidemic effect has not been thoroughly studied.

The present study aimed to investigate eupatilin as a natural antihyperlipidemic agent. We evaluated the HCR-inhibiting activity of eupatilin and investigated its antihyperlipidemic effects. This study suggests eupatilin as an HCR inhibitor.

## 2. Materials and Methods

### 2.1. Materials

**Figure 1:**
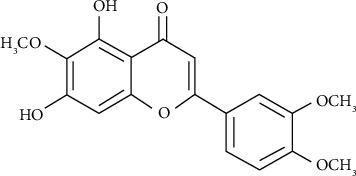
The chemical structure of eupatilin.

Assay kits to evaluate triglyceride (TG), total cholesterol (TC), and high-density lipoprotein (HDL) cholesterol in serum were purchased from Asan Pharmaceutical Co., Ltd. (Gyeonggi-do, South Korea). Eupatilin (Figure 1), lovastatin, Triton WR-1339, cholestyramine, NADPH, RS-HMG-CoA, EDTA-K, dl-dithiothreitol (DDT), and all other chemicals and reagents were purchased from Sigma–Aldrich (St. Louis, MO, USA) unless stated otherwise.

### 2.2. Partial Purification and Activity of HCR

The HCR-inhibitory effect of eupatilin was analyzed according to the previously described method [5, 11, 12]. The rate of NADPH oxidation was determined using HCR isolated from the liver of three 8-week-old male Sprague–Dawley (SD) rats (weight: 250–300 g) stimulated with 5% cholestyramine. To confirm the oxidation rate, a reaction mixture at pH 6.8 containing 0.16 M potassium phosphate, 0.2 M potassium chloride, 1 mM DTT, 0.2 mM NADPH, 4 mM EDTA, and 0.1 M RS-HMG-CoA was used.

### 2.3. Animals

Male ICR mice (weighing 27–29 g), supplied by Orient Bio Inc. (South Korea), were housed in wire cages at 20 ± 5°C and 50 ± 10% humidity, fed standard laboratory chow (Oriental Laboratory Animal Breeding Center, Seoul, Korea), and allowed water was ad libitum. Approval from the Committee for the Care and Use of Laboratory Animals at Eulji University was obtained for all animal experiments, which were conducted in accordance with the guidelines of the Eulji University Institutional Animal Care and Usage Committee (IACUC) (approval no. EUIACUC 21-26).

### 2.4. Induction of Hyperlipidemia in Mice

To confirm the antihyperlipidemic effect of eupatilin, hyperlipidemia was induced in mice using corn oil [13]. Each group consisted of six mice. Corn oil (1 g/kg) was administered orally 2 hours after a single oral treatment of eupatilin (25 and 50 mg/kg) or lovastatin (10 mg/kg). Blood samples were collected by cardiac puncture under anesthesia 2 hours after corn oil administration.

In addition, hyperlipidemia was induced in mice using Triton WR-1339 [5]. Six mice were used for each group. Eupatilin (25 and 50 mg/kg) or lovastatin (10 mg/kg) was orally treated once daily for three days, and Triton WR-1339 was administered through the tail vein 16 hours after the last treatment. Fasting was maintained for 16 hours until administration with Triton WR-1339. Blood samples were collected by cardiac puncture under anesthesia 18 hours after administration of Triton WR-1339.

### 2.5. Determination of Serum TC, TG, and HDL Cholesterol Levels

Serum TC, TG, and HDL cholesterol levels were measured using commercially available assay kits and previously published protocols [14–16]. Serum TC was quantified using an assay kit for measuring TC to which the enzyme method of Allain et al. [14] was applied. 3.0 ml of enzyme solution was added to 0.02 ml of serum, mixed, and reacted at 37°C for 5 minutes. The absorbance of the reactants was measured at a wavelength of 500 nm using a spectrophotometer (BioPhotometer, Eppendorf), and the TC content was quantified by substituting it into a calibration curve. Serum TG was measured using an assay kit for measuring TG according to the principle of the color development method using the enzymatic method of Sardesai and Manning [15]. 3.0 mL of enzyme solution was added to 0.02 mL of serum, mixed, and reacted at 37°C for 10 minutes. TG content was quantified by measuring the absorbance of the reactant at a spectrophotometer (BioPhotometer, Eppendorf) wavelength of 550 nm and substituting it into a calibration curve.

In addition, serum HDL-cholesterol was measured using an assay kit for measuring HDL-cholesterol [16]. 0.2 mL of separation solution was added to 0.2 mL of serum, mixed, and left at room temperature for 10 minutes. 0.1 mL of supernatant obtained by centrifugation at 3,000 rpm for 10 minutes was taken, mixed well with 3.0 mL of enzyme reagent, and reacted at 37°C for 5 minutes. The absorbance was measured at a wavelength of 500 nm with a spectrophotometer (BioPhotometer, Eppendorf), and HDL cholesterol content was quantified by substituting it into a calibration curve.

### 2.6. Statistical Analysis

All the data are expressed as a mean ± standard deviation, and statistical significance was analyzed using one-way ANOVA followed by the Student's *t*-test and the Student–Newman–Kelus test. Statistical significance was set at *p* < 0.05.

## 3. Results

**Figure 2:**
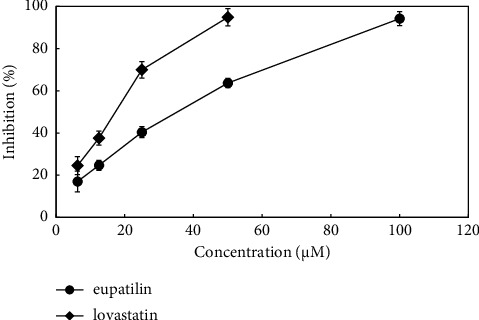
Inhibitory effect of eupatilin on the HMG-CoA reductase activity. Each result represents the mean ± SD of triplicate experiments.

The HCR-inhibiting effect of eupatilin, the main constituent of AP, was evaluated to investigate the antihyperlipidemic effects of this natural herb. Eupatilin potently inhibited HCR activity with an IC_50_ value of 34.2 *μ*M (Figure 2).

**Figure 3:**
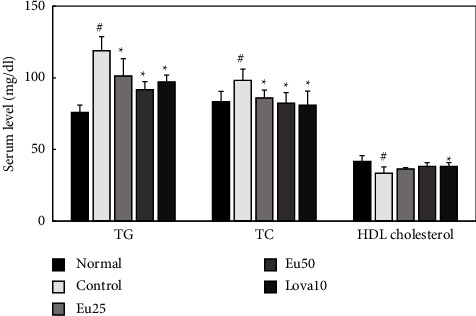
Effects of eupatilin on serum TG, TC, and HDL cholesterol levels in corn oil-induced hyperlipidemic mice. Corn oil was administered orally in the control, Eu25, Eu50, and Lova10 groups. The test agents (control, saline alone; Eu25, 25 mg/kg of eupatilin; Eu50, 50 mg/kg of eupatilin; Lova10; 10 mg/kg of lovastatin) were administered orally 2 h before corn oil-administration. All values are represented as mean ± SD (*n* = 6). #significantly different compared with the normal group (*p* < 0.05) ^*∗*^significantly different compared with the control group (*p* < 0.05).

In addition, because HCR inhibitors, including lovastatin, are used to treat hyperlipidemia, the antihyperlipidemic effects of eupatilin were investigated in corn oil- and Triton WR-1339-induced hyperlipidemic mice. Orally treated eupatilin significantly reduced TC and TG levels in the serum of corn-oil-induced hyperlipidemic mice in a dose-dependent effect compared to those in the control group. Moreover, HDL cholesterol levels improved; however, the result was not significant (Figure 3 and Table 1).

**Figure 4:**
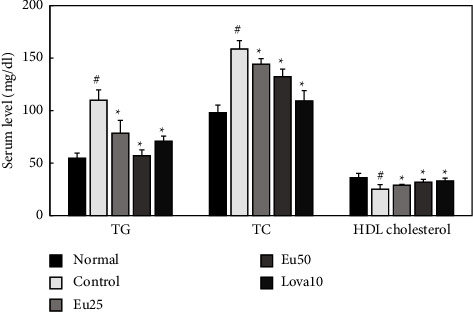
Effects of eupatilin on serum TG, TC and HDL cholesterol levels in Triton WR-1339-induced hyperlipidemic mice. Triton WR-1339 was administered intravenously in the control, Eu25, Eu50, and Lova10 groups. The test agents (control, saline alone; Eu25, 25 mg/kg of eupatilin; Eu50, 50 mg/kg of eupatilin; Lova10; 10 mg/kg of lovastatin) were administered orally once a day for three days. The last oral administration was conducted 16 h before Triton WR-1339 treatment. All values are represented as mean ± SD (*n* = 6). #significantly different compared with the normal group (*p* < 0.05) ^*∗*^significantly different compared with the control group (*p* < 0.05).

Eupatilin also significantly lowered TC and TG levels by 48.1% and 16.6%, respectively, in Triton WR-1339-induced hyperlipidemic mice when administered orally at a concentration of 50 mg/kg. In contrast to corn oil-induced hyperlipidemia in mice, orally administered eupatilin significantly increased HDL cholesterol levels in Triton WR-1339-induced hyperlipidemic mice. Notably, these antihyperlipidemic effects of eupatilin were dose-dependent (Figure 4 and Table 2).

## 4. Discussion

Homeostasis is important for lipid metabolism, and hyperlipidemia develops when homeostasis is disrupted. The importance of HCR as a therapeutic target for hyperlipidemia has been demonstrated in several studies. HCR, which acts as a rate-limiting enzyme in the process of cholesterol biosynthesis from acetate, is typically inhibited by statins, which have been long used as a treatment for hyperlipidemia [4, 5]. Statins are generally safe drugs; however, they are related with several side effects such as muscle pain [17] and the risk of diabetes [18]. In addition, the therapeutic effect of statin may be limited in certain age groups [19]. Herbal medicines have gained increasing attention for their antihyperlipidemic effects as safe and highly effective drugs to replace statins. We have previously analyzed the antihyperlipidemic effect of natural ingredients with NF-*k*B inhibitory effects by reversely interpreting the results of studies showing that statins exhibit anti-inflammatory effects by inhibiting NF-*κ*B inhibition [6–8].

Eupatilin, the main component of AP, is widely used to treat gastric inflammation in East Asia. Eupatilin exhibits anti-inflammatory effects by inhibiting NF-*κ*B [1]. Here, we evaluated the HCR-inhibiting and antihyperlipidemic effects of eupatilin. We revealed that eupatilin effectively inhibited HCR and significantly reduced serum TC and TG levels in hyperlipidemic mouse models, demonstrating its capability as a curative agent for hyperlipidemia. However, eupatilin showed a differential HDL cholesterol-enhancing effect in Triton WR-1339- and corn oil-induced hyperlipidemic mice. Triton WR-1339 induces oxidative reactions and prevents the catabolism of triacylglycerol-rich lipoproteins by lipoprotein lipase (LPL) to induce hyperlipidemia [20]. In contrast, corn oil acts as a fat source when administered to animals [21], and the transient increase in this fat is thought to induce hyperlipidemia. In addition to NF-*k*B inhibition, eupatilin exhibits anti-inflammatory effects through antioxidant effects [22]; therefore, it exhibits a superior effect in recovering HDL cholesterol levels in Triton WR-1339-induced hyperlipidemia mice that exhibit oxidative action.

This study demonstrates that eupatilin can reduce hyperlipidemia by inhibiting HCR activity. However, long-term administration experiments, toxicity tests, and clinical trials are needed to confirm the therapeutic potential of eupatilin in treating hyperlipidemia.

## 5. Conclusion

To discover a natural component that suppresses hyperlipidemia, the effect of eupatilin, the main component of AP widely used in inflammatory diseases in East Asia, was confirmed. Eupatilin, which inhibits HCR, lowered TC and TG in mice induced with hyperlipidemia using corn oil or Triton WR-1339. In addition, HDL cholesterol, lowered by Triton WR-1339, was increased by eupatilin. In conclusion, eupatilin exhibits antihyperlipidemic effects through HCR inhibition.

## Figures and Tables

**Table 1:** Effects of eupatilin on serum TG, TC, and HDL cholesterol levels in corn oil-induced hyperlipidemic mice.

Group	Dose (mg/kg)	Serum level (mg/dl)		
		TG	TC	HDL cholesterol
Normal	—	75.93 ± 10.11^*a*^	83.42 ± 7.97^*a*^	41.75 ± 3.37^*c,d*^
Control	—	119.07 ± 9.49^*d*^	98.20 ± 8.20^*d*^	33.58 ± 3.37^*a*^
Eupatilin	25	101.27 ± 5.45^*b*^	86.07 ± 12.97^*a,b*^	36.57 ± 3.36^*a,b*^
	50	91.82 ± 8.19^*c*^	82.42 ± 5.62^*a,b,c*^	38.27 ± 8.28^*a,b,c,d*^
Lovastatin	10	97.07 ± 11.44^*b*^	80.97 ± 4.84^*a,b,c*^	38.22 ± 2.97^*b,c*^

The serum levels are expressed as the mean ± S.D, (*n* = 6). ^a,b,c,d^Items with the same letter in each column were not significantly different (*p* < 0.05).

**Table 2:** Effects of eupatilin on serum TG, TC, and HDL cholesterol levels in Triton WR-1339-induced hyperlipidemic mice.

Group	Dose (mg/kg)	Serum level (mg/dl)		
		TG	TC	HDL cholesterol
Normal	—	54.68 ± 4.97^*a*^	98.13 ± 7.21^*a*^	36.22 ± 4.00^*c*^
Control	—	109.97 ± 9.68^*c*^	158.75 ± 7.93^*b*^	25.38 ± 4.25^*a*^
Eupatilin	25	78.52 ± 12.22^*b*^	144.23 ± 5.30^*c*^	29.05 ± 0.85^*d*^
	50	57.08 ± 5.55^*a*^	132.35 ± 7.19^*d*^	31.97 ± 2.53^*b*^
Lovastatin	10	70.97 ± 4.76^*b*^	109.18 ± 9.78^*e*^	33.07 ± 2.68^*b,c*^

The serum levels are expressed as the mean ± S.D, (*n* = 6). ^a, b, c ,d, e^Items with the same letter in each column were not significantly different (*p* < 0.05).

## Data Availability

The data used to support the findings of this study are available from the corresponding author upon request.
